# Comparison of the anesthetic effect of alkalized lidocaine versus non-alkalized lidocaine administered at a slow rate in mandibular primary molars

**DOI:** 10.34172/joddd.2023.37014

**Published:** 2023-04-03

**Authors:** Karen Torres-Rojas, Victor Chumpitaz-Cerrate, Lesly Chávez-Rimache, Daniella Núñez-Díaz, Vanessa Pérez-Jimenez

**Affiliations:** ^1^Department of Dentistry, Universidad Nacional Mayor de San Marcos, Lima, Peru; ^2^Group Investigación en Ciencias Básicas Estomatológicas (ICBEST), Universidad Nacional Mayor de San Marcos, Lima, Peru; ^3^Pharmacology Laboratory, Universidad Científica del Sur, Lima, Peru

**Keywords:** Dental anaesthesia, Pediatric dentistry, Buffered, Dental pulp diseases, Lidocaine

## Abstract

**Background.:**

There are several invasive dental procedures that require local anesthetics. However, its infiltration is usually associated with anxiety and fear, increasing the perception of pain in pediatric patients. For this reason, it is important to evaluate different strategies for its application. We compared the anesthetic effect of the administration of 2% lidocaine with epinephrine 1:80000 non-alkalized at slow speed and alkalized at fast speed to block the inferior alveolar nerve in deciduous molars.

**Methods.:**

A crossover clinical trial was carried out whose sample consisted of 38 patients between 6-10 years who required bilateral pulp treatment in their first mandibular primary molars. At the first appointment, they received 2% lidocaine with 1:80000 alkalinized epinephrine administered at a fast rate, and at the second appointment, 2% lidocaine with 1:80000 non-alkalized epinephrine administered at a low speed. We evaluated the onset of action, duration of the anesthetic effect, and intensity of pain during its infiltration.

**Results.:**

We found that non-alkalized lidocaine at slow speed had a shorter onset time of action (57.21±22.21 seconds) and longer duration of effect (170.82±43.75 minutes) compared to administration of alkalinized lidocaine at fast speed (74.03±22.09 seconds, 148.24±36.24 minutes, respectively). There was no difference in the level of pain intensity.

**Conclusion.:**

In this study, the slow administration of the non-alkalized local anesthetic showed a shorter onset time of action and a longer duration of the anesthetic effect in comparison with the alkalized local anesthetic administered at a rapid rate in the blockade of the inferior alveolar nerve in deciduous molars.

## Introduction

Local anesthetics prevent pain impulse conduction during several invasive dental procedures, such as pulp treatments, extractions, etc. However, the infiltration of these drugs produces pain, and its perception is greater in pediatric patients.^1-3^ This pain in turn produces anxiety in children and parents, which frequently leads to non-compliance with visits to the pediatric dentist.^4^

Regarding the mechanism of action of local anesthetics, they come in two forms: ionized and non-ionized. The non-ionized form, due to its liposolubility, easily crosses the neuronal membrane and concentrates in the cytosol, where it is ionized to block sodium channels. By blocking sodium channels, the perception and conduction of pain are prevented. Therefore, the action of local anesthetics depends on the amount of the ionized form; and this, in turn, is related to their dissociation potential (pKa).^5^

The pKa is the pH at which the local anesthetic is 50% ionized and 50% non-ionized. The pKa of local anesthetics varies between 7.9 and 8.9.^6^

In inflammatory and infectious processes, the medium acidifies, which decreases the non-ionized form’s formation, reducing the efficacy of the local anesthetic.^7-10^ In addition, the pain caused by the application of local anesthetics with vasoconstrictor could be greater because they have a lower pKa.^11^ For this reason, several strategies have been proposed to reduce pain during the administration of local anesthetics, including slow-speed application and alkalinization.^12-14^ Alkalinization consists of adding a buffer to the local anesthetic, increasing its pKa, and approaching tissue pH (7.4). This could decrease the pain during the application, reduce onset time and increase the duration of action of local anesthetics.^9,14-16^ A recent systematic review by Tirupathi et al reported that pain scores were lower in children who were administered the alkalinized local anesthetic by blocking the inferior dental nerve. Nonetheless, the current evidence is limited, and quality studies are needed to evaluate the efficacy of this intervention.^17^

Based on this, considering that the administration of local anesthetics (especially lidocaine with epinephrine as it is the most used) is a necessary procedure to perform several dental treatments in pediatric patients.^15,18-20^ The study aimed to compare the anesthetic effect of the administration of lidocaine 2% with epinephrine 1:80 000 non-alkalinized at a slow speed and alkalinized at a fast speed for the blockade of the inferior dental nerve in deciduous molars.

## Materials and Methods

###  Study setting and design

 An experimental, longitudinal, prospective study was conducted. The sample consisted of 33 patients aged 6 to 10 years ASA I (American Society of Anesthesiologists category 1) who required pulp treatment and were enrolled from August to October 2019 in the Pediatric Dentistry Service of the Hospital Nacional Arzobispo Loayza (HNAL).

###  Ethical aspects

The CONSORT guidelines for randomized clinical trials were followed for the reporting of the research. The study was approved by the ethics committee of the Faculty of Medicine of the Universidad Nacional Mayor de San Marcos (19-0058) and the Hospital Nacional Arzobispo Loayza (HNAL: 112-2019). In addition, the study was conducted under The International Conference on Harmonization Good Clinical Practice guideline (ICH-GCP).^21^ On the other hand, mothers who accepted their children’s participation in the study signed an informed consent form and their children signed the informed assent.

###  Participants

For the calculation of the sample size, we used Epidat v4.2 software (https://www.sergas.es) for the difference of means of paired groups. We considered a mean difference to detect of 0.55 and a standard deviation of differences of 0.73 with a statistical power of 80% and a significance level of 5%.^9^ The sample size was 32 patients, therefore a sample size of 33 patients was considered in our study.

Inclusion criteria were patients between 6 and 10 years of age ASA I requiring pulp treatment in the first two molars of both hemi-mandibles, classified as positive or definitely positive according to the Frankl scale.^22^ Patients with dentoalveolar abscesses or chronic infectious conditions, patients with teeth requiring pulpotomies, and patients with a history of allergic reactions to any component of the local anesthetic cartridge were excluded.

 Coordination was made with the person responsible for the pediatric dentistry service of the HNAL and a plan for selecting or recruiting participants was approved. An investigator was in the pediatric dentistry office and assessed compliance with the eligibility criteria of patients attending their appointment during the day. If the pediatric patient met the eligibility criteria, parents or guardians were consulted and the benefits/risks of the study were explained to them via the informed consent form as to whether the patient could participate in the study. In addition, the child explained the procedure using an informative portfolio and was given informed assent.

 Subsequently, the treatment assignment was concealed by using opaque envelopes (red and blue). In which the pediatric patient had to choose one of the envelopes indicating the treatment assigned for the first appointment (alkalinized local anesthetic administered at a fast speed). Then on the second appointment, the other treatment was administered (non-alkalinized local anesthetic administered at a slow speed).

 Moreover, both the patient and the parent were unaware of the type of technique administered (the same local anesthetic was used for both groups). All local anesthetic administrations were performed by a single operator. After pulp treatment, all patients have prescribed ibuprofen suspension at a dose of 20mg/kg every 8 hours (100 mg/5 mL) after the anesthetic effect had ended.

###  Preparation of local anesthetics

For alkalinization of lidocaine 2% with adrenaline 1:80 000 with sodium bicarbonate, the technique performed by Saatchi et al^23^ was followed in which 0.18 mL of the anesthetic solution was extracted from the cartridge with a tuberculin syringe and that volume was discarded. Then 0.18 mL of an ampoule of 8.4% sodium bicarbonate was loaded and added to the cartridge. Subsequently, the solution was slowly shaken 20 times and once the preparation was finished, the anesthetic was immediately administered with a cartridge syringe.

 The administration of the alkalized local anesthetic was carried out at a rapid speed in a period of 15 seconds, marking the cartridge in 3 segments of 17 mm each and each segment was administered in 5 seconds controlled by a stopwatch.

 The administration of the non-alkalized local anesthetic was carried out at a slow speed for a period of 60 seconds, for this, the cartridge was marked in 6 segments of 8.5 mm each and each segment was administered in 10 seconds controlled by a stopwatch.

###  Outcomes

 The onset of action of the local anesthetic was recorded by the elapsed time in seconds. From the administration of the local anesthetic until the patient presented a tingling sensation on the tip of the tongue and/or lower hemilabium on the side where the administration of the local anesthetic.

 To evaluate the duration of the anesthetic effect, the parent or guardian of the child was asked to remain in the waiting room so that the researcher could register the duration of the anesthetic effect. This duration of the anesthetic effect was registered by the time elapsed in minutes. From the onset of action until the disappearance of the numbness sensation of the tip of the tongue and/or lower hemilabium on the side of the administration of the local anesthetic.

For the evaluation of pain intensity, the Wong-Baker Scale known as the verbal graphic scale was used. According to the systematic review by Stinson et al,^19^ this scale presents adequate validity and reliability for the evaluation of pain in pediatric patients. This scale consists of a numerical vertical line (from 0 to 10) and six faces with different expressions of pain. In which an increasing discomfort is shown from a face without pain to an image in which the maximum expression of pain appears. The children registered on the card the intensity of pain they perceived during the inoculation of the anesthetic solution. All patients were fitted with glass ionomer as a final restoration.

###  Statistical analysis

 Stata software version 16.0 was used for statistical analysis. Numerical variables were expressed as a mean and standard deviation; categorical variables were expressed as absolute and relative frequencies. In addition, the intention-to-treat analysis was performed considering the last measurement registered. For the evaluation of categorical variables, the chi-square test was used. For numerical variables, the Student’s t-test for paired samples was used. Considering a significance level of 5% to reject the two-tailed null hypothesis.

## Results

 Of the 47 patients who met the eligibility criteria, 14 patients were excluded. Of the total 33 patients included, 17 were male and 16 were female with a mean age of 7.09 ± 1.48 years. Patients’ mandibular first molars were randomized to the alkalinized lidocaine group administered at a fast speed (n = 33) and the non-alkalinized lidocaine group administered at a slow speed (n = 33).

**Figure 1:**
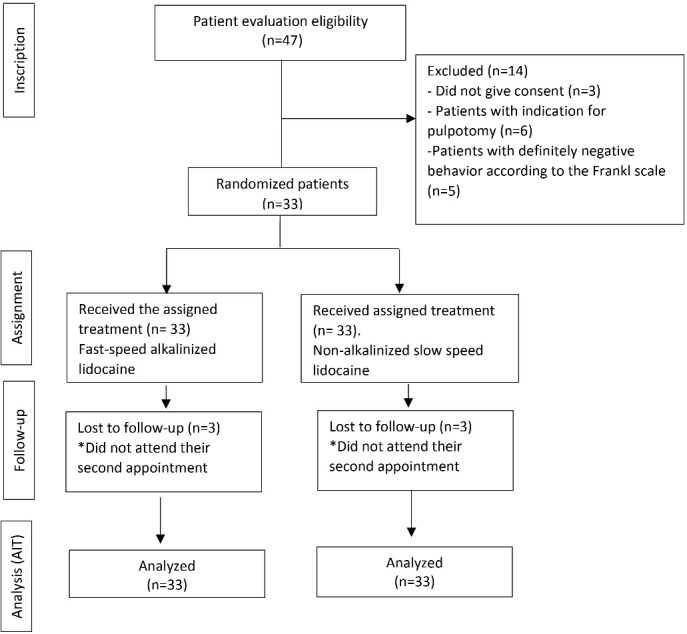
Flow chart of the participants in the clinical trial according to CONSORT. *Note*. AIT: Intention-to-treat analysis

In the follow-up, three patients did not attend their second appointment. Therefore, a total of 63 injections were administered using the inferior dental nerve block technique. The flow diagram of the participants is presented in Figure 1.

###  Onset of anesthesia

**Figure 2:**
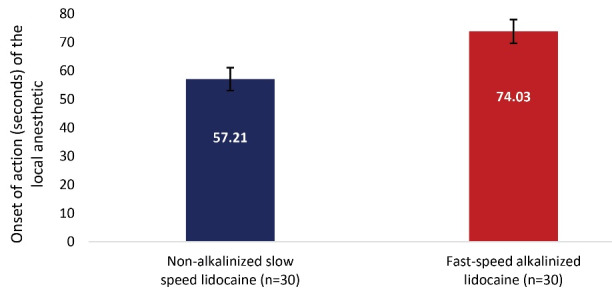
Onset of action (seconds) according to the study groups

Non-alkalinized lidocaine administration at a slow speed produced a shorter time to onset of action (57.21 ± 22.21 seconds [95% CI: 52.63 to 63.79]) compared to alkalinized lidocaine administration at a fast speed (74.03 ± 22.09 seconds [95% CI: 68.60 to 79.46]) (*P* = 0.021; Figure 2).

###  Duration of anesthesia

**Figure 3:**
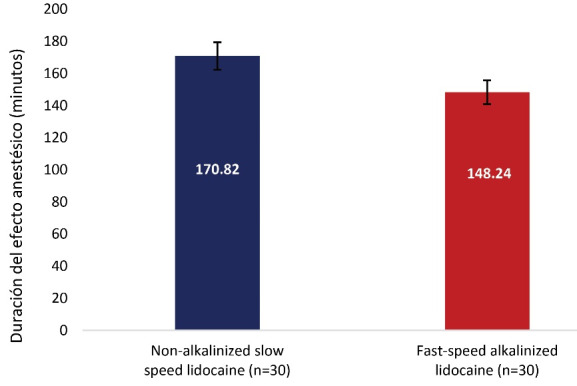
Duration of anesthetic effect (minutes) according to the study groups

Administration of non-alkalinized lidocaine at slow speed produced a longer duration of effect (170.82 ± 43.75 minutes [95% CI: 160.06 to 181.58]) compared to administration of alkalinized lidocaine at fast speed (148.24 ± 36.24 minutes [95% CI: 139.33 to 157.14]) (*P* = 0.041; Figure 3).

###  Assessment of pain intensity according to the visual graphic scale

We found that the majority of patients experienced mild pain in both the group that received non-alkalinized lidocaine at a slow speed and the group that received alkalinized lidocaine at a fast speed. However, there was no significant difference in the intensity of pain between the two study groups. Table 1.

**Table 1:** Pain intensity according to the verbal graphic scale

**Pain intensity level (VGS)**	**Non-alkalinized lidocaine**		**Alkalinized lidocaine**		***P*** value*
	**No.**	**%**	**No.**	**%**	
No pain	3	25.0	9	75.0	0.188
Little pain	17	51.5	16	48.5	
Moderate pain	10	76.9	3	23.1	
Severe pain	1	33.3	2	66.7	
Very severe pain	1	33.3	2	66.7	
Unbearable pain	1	50.0	1	50.0	

*Note.* VGS: verbal graphic scale. *Chi-square test.

## Discussion

 It was found that the administration of non-alkalinized lidocaine at a slow speed presented a shorter time of onset of action and a longer duration of effect of the local anesthetic, compared to the group that received the administration of alkalinized lidocaine and administered at a fast speed. However, the intensity of pain from the injection of the anesthetic was similar in both groups.

###  Onset of action

It was found that the administration of lidocaine not alkalinized and administered at a slow speed (57.21 ± 22.21 seconds) presented a shorter onset of action time, compared to the administration of lidocaine alkalinized and administered at a fast speed (74.03 ± 22.09 seconds) (*P* = 0.021). These results differ from those reported by Chumpitaz-Cerrate et al^24^ who found that the time to onset of action was shorter in the alkalinized lidocaine group (105.72 ± 9.7 seconds) compared to the non-alkalinized lidocaine group (157.52 ± 12.1 seconds) (*P* = 0.002). Nonetheless, the study by Hobeich et al^25^ found that the time to onset of action was similar in patients who were administered 5% alkalinized lidocaine (116 ± 68 seconds), 10% (121 ± 59 seconds); and those who were administered non-alkalinized lidocaine (119 ± 68 seconds) in healthy maxillary canines. These differences could be due to variations in methodology and participant characteristics. Regarding the former, different ways of assessing the onset of anesthesia such as electrical pulp testing and palpation were used. In addition, the studies had different designs such as parallel and crossover randomized clinical trials. On the other hand, different administration times and anesthetic techniques were used. Hobeich et al^25^ used the infiltration technique to anesthetize the upper teeth; this makes a difference because the cortex in the mandible is thicker, denser, and has less porosity in comparison with the maxillary bone.^26^

Regarding clinical characteristics, the studies by Hobeich et al^25^ and Chumpitaz-Cerrate et al^24^ were performed in healthy adult patients and our study was in children, so the degree of anxiety before these procedures is higher in children.^27^

Other studies such as that of Chopra et al^28^ reported that the onset time of local anesthetic (lidocaine 2% with epinephrine 1:200 000) in pediatric patients was 86 ± 27.8 seconds, while for alkalinized lidocaine it was 84.2 ± 28.9 seconds (*P* = 0.824). On the other hand, Kurien et al^15^ conducted a study on a pediatric population aged 6 to 12 years, who required bilateral pulp therapy in primary mandibular molars. They found that rapid administrations (30 to 40 seconds) of alkalinized and preheated anesthetics had a shorter time to onset of action (130 seconds) compared to anesthetics administered at a slow speed (60 seconds).

These differences compared to our study could be because different concentrations of adrenaline (lidocaine 2% with epinephrine 1:200 000) and sodium bicarbonate were used to alkalinize the anesthetic solution^15^ using a dilution of 1:10). In addition, the pain was assessed every 15 seconds with gingival probing. Unlike our study, it was assessed with the appearance of a tingling sensation in the tongue and lower lip.

###  Duration of anesthetic effect

It was found that the administration of non-alkalinized lidocaine at a slow speed (170.82 ± 43.75 minutes) presented a longer duration of anesthetic effect, compared to the administration of alkalinized lidocaine at a fast speed (148.24 ± 36.24 minutes) (*P* < 0.05). Similarly, Kurien et al^15^ found that fast-speed (30 to 40 seconds) administrations of alkalinized anesthetic and preheated anesthetic (30 to 40 seconds) presented a shorter duration of effect, compared to slow administration of anesthetic (60 seconds) (*P* < 0.001). This could be because the anesthetic solution when administered at a slow speed, allows the solution to remain in contact with the nerve fibers for a longer time. This would lead to an increase in the duration of the time effect of the local anesthetic. On the other hand, Chumpitaz-Cerrate et al^24^ reported that the duration of effect was similar between the administration of alkalinized (194.44 ± 8.5 minutes) and non-alkalinized (205.4 ± 11.6 minutes) lidocaine in adult patients. These reported differences may be due to the clinical characteristics of the participants and the different alkalinization methods.

###  Pain during injection

Previous studies have reported that the application of alkalinized local anesthetic reduces injection pain during its application compared to non-alkalinized local anesthetic. In medicine, the alkalinization of local anesthetics has been widely used to achieve greater success in dental anesthesia. The local anesthetic solution has an acidic nature, which can produce pain during infiltration and delay the onset of anesthesia.^29^ Adding sodium bicarbonate to the local anesthetic increases the alkalinity of the solution, potentially allowing for a shorter time to onset of action of the anesthetic.^7,9,14,15^ However, the present investigation found that rapid administration of alkalinized lidocaine had similar pain intensity during application compared to slow administration of non-alkalinized lidocaine. This was similar to that reported by Burns et al^30^ who reported that intradermal administration of 1% lidocaine with alkalinized epinephrine 1:100 000 (according to the visual analog scale [VAS]: 18.3 ± 20.3) presented similar pain to the administration of non-alkalinized lidocaine (23.5 ± 19.1). Schellenberg et al^31^ found that, in adult patients with symptomatic irreversible pulpitis in lower premolars or molars, non-alkalinized 4% lidocaine with epinephrine 1:100 000 had similar anesthetic success as alkalinized lidocaine (40% [95% CI: 26.4-54.9]) and 32% [95% CI: 19.5-46.7], respectively. Success was defined as no or mild pain (VAS ≤ 54 mm) during root canal access or instrumentation. Chopra et al^28^ performed a randomized crossover clinical trial and found that alkalinized solution did not reduce pain from infiltration. In addition, Baker et al^32^ conducted a randomized crossover clinical trial in 25 children (10 to 12 years) and found that there was no difference in infiltration pain, the onset of action, and duration of anesthetic between the group administered alkalinized and non-alkalinized anesthetic solutions.

On the other hand, Kurien et al^15^ found that fast-speed (30 to 40 seconds) administrations of alkalinized anesthetic and preheated anesthetic (30 to 40 seconds) produced less pain intensity during local anesthetic injection compared to anesthetic administered at slow speed (60 seconds) (*P* < 0.001). Similarly, systematic reviews such as Hanna et al^13^ reported that alkalinized local anesthetics reduced pain (Mean difference: -1.17 [95% CI: -1.68 to -0.67]) during their administration compared to non-alkalinized local anesthetics. In addition, Tirupathi et al^17^ found that alkalinized local anesthetic reduced pain perception compared to non-alkalinized local anesthetic (Mean difference: -0.32 [95% CI: -0.55 to -0.09]). Similarly, Kattan et al^14^ reported that alkalinized local anesthetics improved the success of anesthesia (variably defined using the verbal graphic scale, visual analog scale, and a cold test or pulpal electrical tests) compared with non-alkalinized local anesthetics (OR: 2.29 [95% CI: 1.11 to 4.71]).

 This variability in results is due to high methodological heterogeneity (different study designs, methods for alkalinization of local anesthetics, different presentations of local anesthetic and buffer, and different pain scales) and clinical heterogeneity (different diagnoses, characteristics of the study populations such as age, sex, oral status, different injection speed of the anesthetic and the presence of individual pain thresholds).

This study had some limitations; first, pain perception is subjective and may vary among patients; however, a validated scale for pain assessment in pediatric patients, such as the Wong-Baker visual analog scale, was used.^19,20,33^

## Conclusion

 According to our study, the slow administration of lidocaine 2% with non-alkalinized epinephrine 1:80 000 has a shorter time to onset of action and longer duration of anesthetic effect compared to the rapid administration of lidocaine 2% with alkalinized epinephrine 1:80 000 in lower dental nerve block in deciduous molars. However, injection pain was similar between both study groups.

## Acknowledgments

 We are grateful to the patients who participated in the study and contributed substantially to the research.

## Competing Interests

 There is no conflict of interest among all authors.

## Ethical Approval

 The original certificates issued by the Institutional Research Ethics Committee of the Hospital Nacional Arzobispo Loayza and the Research Ethics Committee of the Department of Medicine of the Universidad Nacional Mayor de San Marcos are presented.

## Funding

 Self-financed.
